# Uterine Artery Embolization and Double-J (DJ) Stenting in a Case of Urinary Retention Due to a Massive Cervical Fibroid: A Case Report

**DOI:** 10.7759/cureus.30013

**Published:** 2022-10-06

**Authors:** Hard Tilva, Surekha Tayade, Nidhi Makhija, Arzoo Chadha

**Affiliations:** 1 Department of Obstetrics and Gynaecology, Jawaharlal Nehru Medical College, Datta Meghe Institute of Medical Sciences (Deemed to be University), Wardha, IND

**Keywords:** hysterectomy, dj stenting, uterine artery embolization (uae), urinary retention, cervical fibroid

## Abstract

In women, the most common solid tumor of the pelvis is a uterine fibroid. A large cervical fibroid can also cause urinary incontinence in women. We report a case of a 45-year-old woman with urinary retention that was initially diagnosed as an anterior wall uterine fibroid in the peripheral health center but turned out to be a massive cervical fibroid. After the initial evaluation, it was determined that the cervical fibroid was huge and impacted the pelvis, and there was a possibility of a torrential operative hemorrhage. Thus, preoperative uterine artery embolization (UAE) was performed to prevent intraoperative blood loss, and Double-J (DJ) stenting was performed to avoid ureteric injury. This was followed by a total abdominal hysterectomy, without facing any intraoperative complications. This case demonstrates the importance of proper clinical assessment and the use of skilled interventional radiology procedures such as UAE and DJ stenting in the treatment of a massive cervical fibroid.

## Introduction

The uterine fibroid is the most common solid tumor of the pelvis in women. However, cervical fibroid, which arises from the cervix rather than from the uterus itself, is a very rare entity, and its incidence is less than 1% [1]. The classification of fibroids depends upon where they are situated: centrally, laterally, anteriorly, or posteriorly. They are further classified based on the depth of invasion as interstitial, subserosal, and submucosal polypoids [2]. Risk factors for the development of cervical tumors are almost identical to those of uterine fibroids, which can be due to genetic susceptibility, exposure to steroidal hormones, or various environmental factors. The presenting complaints of the patient could be abnormal uterine bleeding, pain in the abdomen, pressure symptoms such as urinary retention or constipation, or a palpable mass in the abdomen that could be mimicking an ovarian mass. Clinical examination and ultrasound are the mainstays for the diagnosis of cervical fibroids, but magnetic resonance imaging (MRI) is the recommended imaging modality for the characterization of fibroids and to determine their precise anatomical location and size [3]. A qualified interventional radiologist performs a percutaneous, image-guided technique called uterine artery embolization (UAE). It entails inserting a catheter into the femoral artery and injecting embolic material (polyvinyl alcohol particles or gel foam) into both uterine arteries until the flow turns sluggish [4]. The role of uterine artery embolization in our case was to reduce intraoperative blood loss. The best practices for preventing ureteric damage include preoperative stenting, intraoperative ureter delineation, and dissection inside the fibroid capsule. Exploratory laparotomy is the mainstay of treatment for massive fibroids, and due to their close relationship with the bowel, bladder, and ureters, injury to them is a major surgical obstacle.

## Case presentation

A 45-year-old multiparous woman presented with complaints of dull, aching abdominal pain for the past four months, as well as a palpable lump in each abdomen. Gradually, the patient also started to complain about the difficulty in micturition associated with a burning sensation and a feeling of incomplete evacuation after passing urine. On clinical examination, there was a palpable lump per abdomen, corresponding to 28-30 weeks of a gravid uterus, that was non-tender, firm in consistency, and had limited mobility. During the speculum examination, the cervix was completely pulled up and there was no bleeding through the ostium (OS). On vaginal examination, the cervix was completely flushed with the mass and could not be felt separately, with no bleeding on touch and fullness of the bilateral fornices and Douglas pouch. Ultrasonography (USG) of the abdomen and the pelvis was suggestive of bilateral hydronephrosis and a large heterogeneous, hypoechoic lesion of 22x17x14.5 cm in the uterus, reaching up to the umbilicus, with another similar lesion seen adjacent to it of size 3.6x2 cm, suggestive of a fibroid.

**Figure 1:**
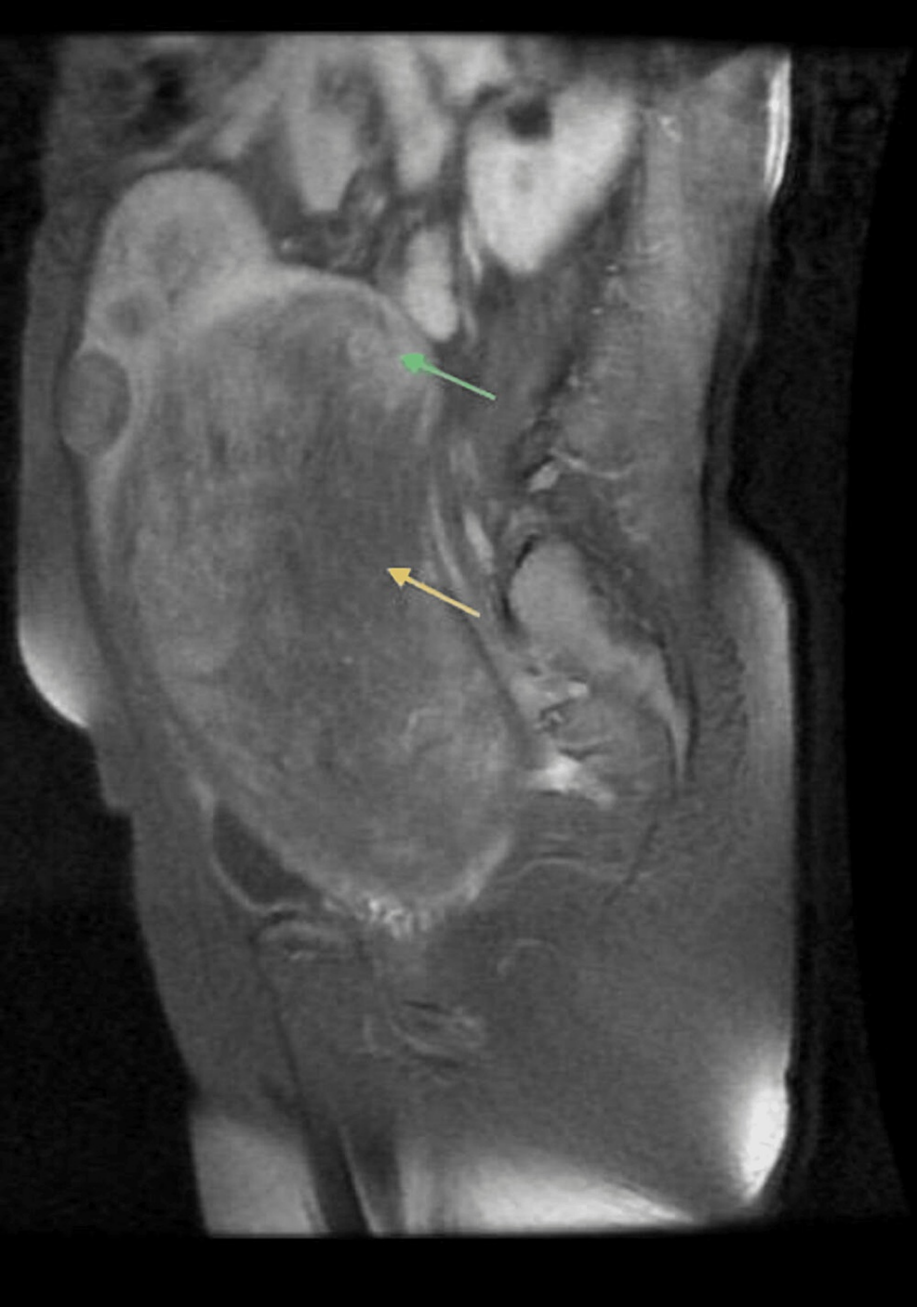
A T1-weighted MRI image with contrast shows an enhancing large mass (green arrow) and necrotic area (yellow arrow) in the uterine posterior wall.

**Figure 2:**
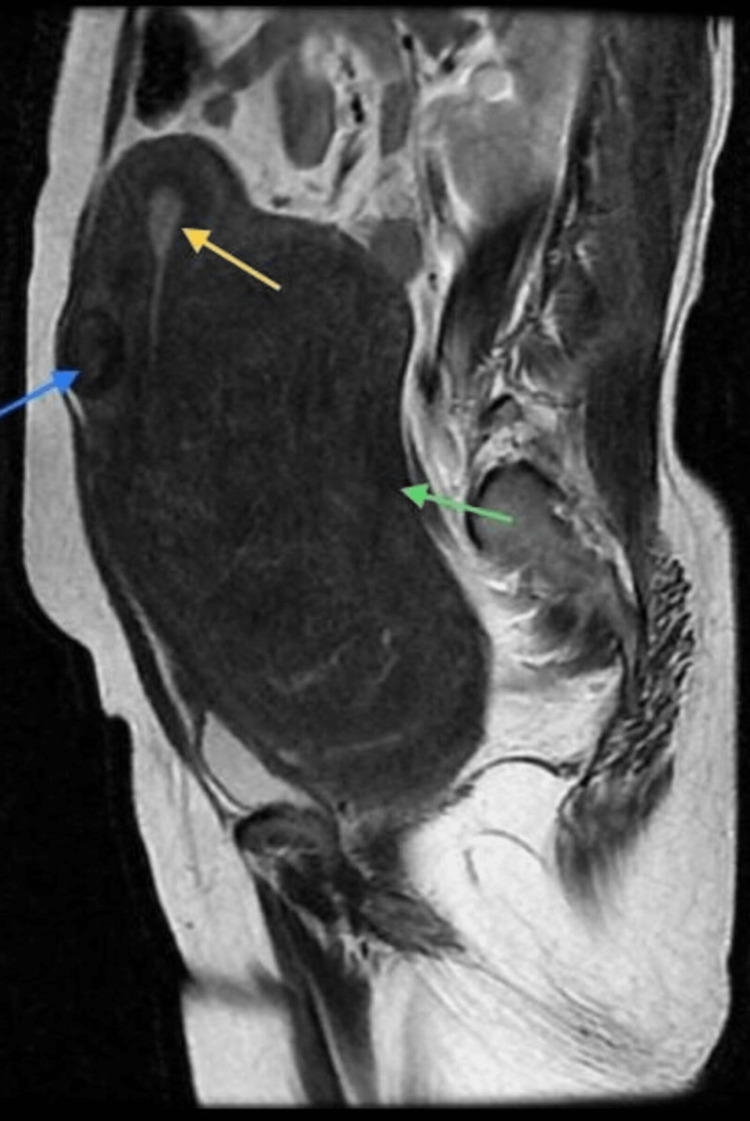
A large posterior wall uterine fibroid (green arrow) displacing endometrium anteriorly (yellow arrow) and a small anterior wall fibroid (blue arrow) on T2-weighted MRI.

A contrast-enhanced magnetic resonance imaging (MRI) of the pelvis was done to evaluate the massive uterine lesion, and midsagittal sections of T1-weighted contrast-enhanced images were suggestive of a large intramural fibroid in the uterus measuring 19x16x10 cm (Figure 1 and Figure 2), which was causing a mass effect in the form of displacement of the bowel loops superiorly and the bladder inferiorly with compression of the bilateral ureters, and a small 2.8x2.2 cm fibroid on the anterior surface of the uterus.

**Figure 3:**
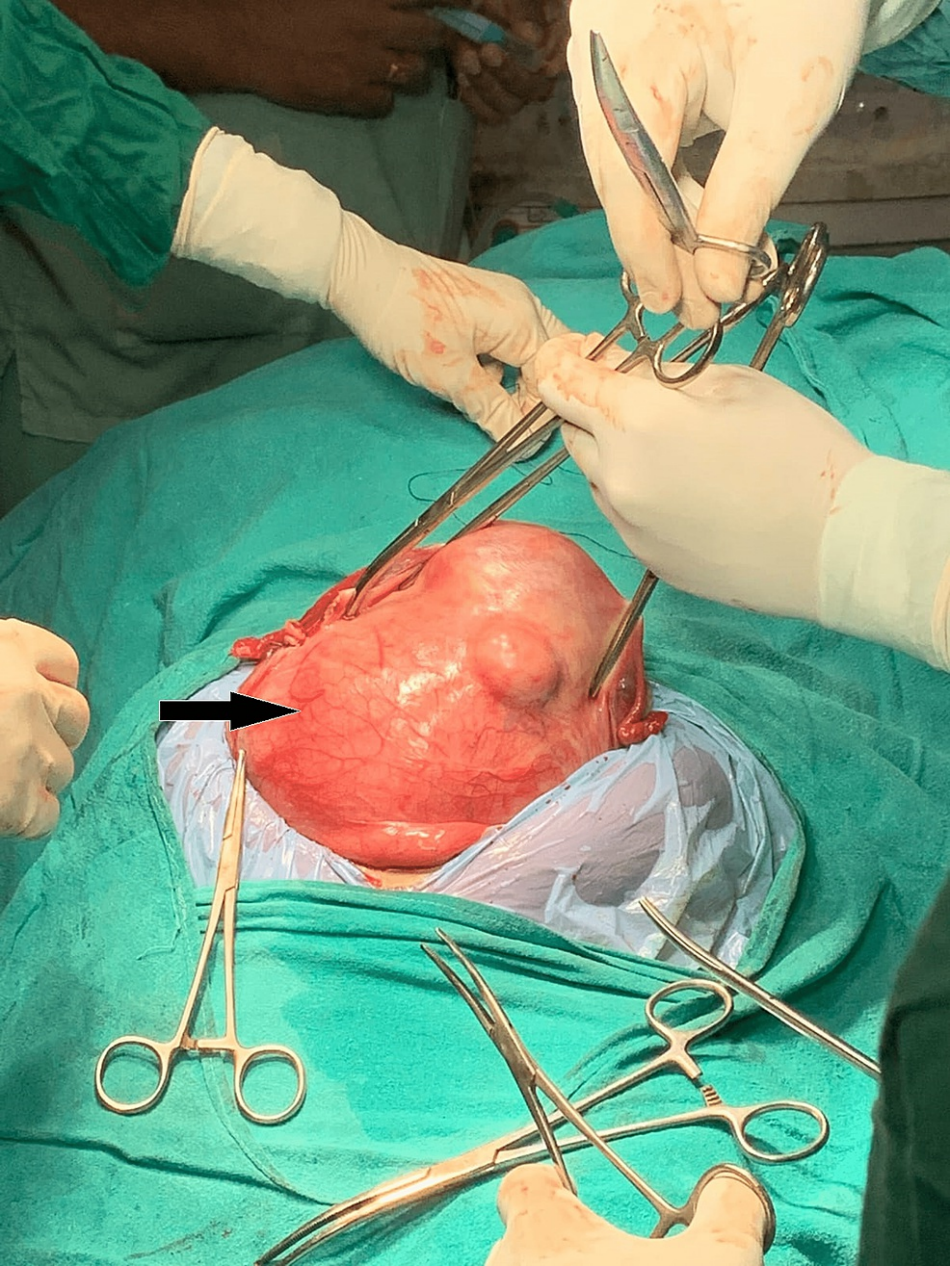
An intraoperative picture showing a massive cervical fibroid arising from the posterior lip of the cervix (black arrow).

A multidisciplinary approach was taken for further management of the case. Preoperative uterine artery embolization was done by cannulation of the bilateral internal iliac arteries with selective cannulation of the uterine artery using the Progreat microcatheter (from Terumo Interventional Systems), and polyvinyl alcohol (PVA) particles of sizes 100-300 nm were used to embolize the tumor blush. After taking informed, valid written consent for double-J (DJ) stenting and exploratory laparotomy with a total abdominal hysterectomy and bilateral salpingo-oophorectomy, on-table per-urethral cystoscope-guided bilateral stenting of both kidneys was done using a 16-cm-size silicone double-J stent before proceeding further with exploratory laparotomy. We decided to perform a laparotomy after 72 hours after uterine artery embolization. On laparotomy, both ovaries were healthy. The uterus had a small intramural fibroid on the anterior wall of the uterus. Only one unit of blood transfusion was needed intra-operatively. The presumed large fibroid on the posterior wall of the uterus was actually a massive cervical fibroid arising from the posterior lip of the cervix (Figure 3).

**Figure 4:**
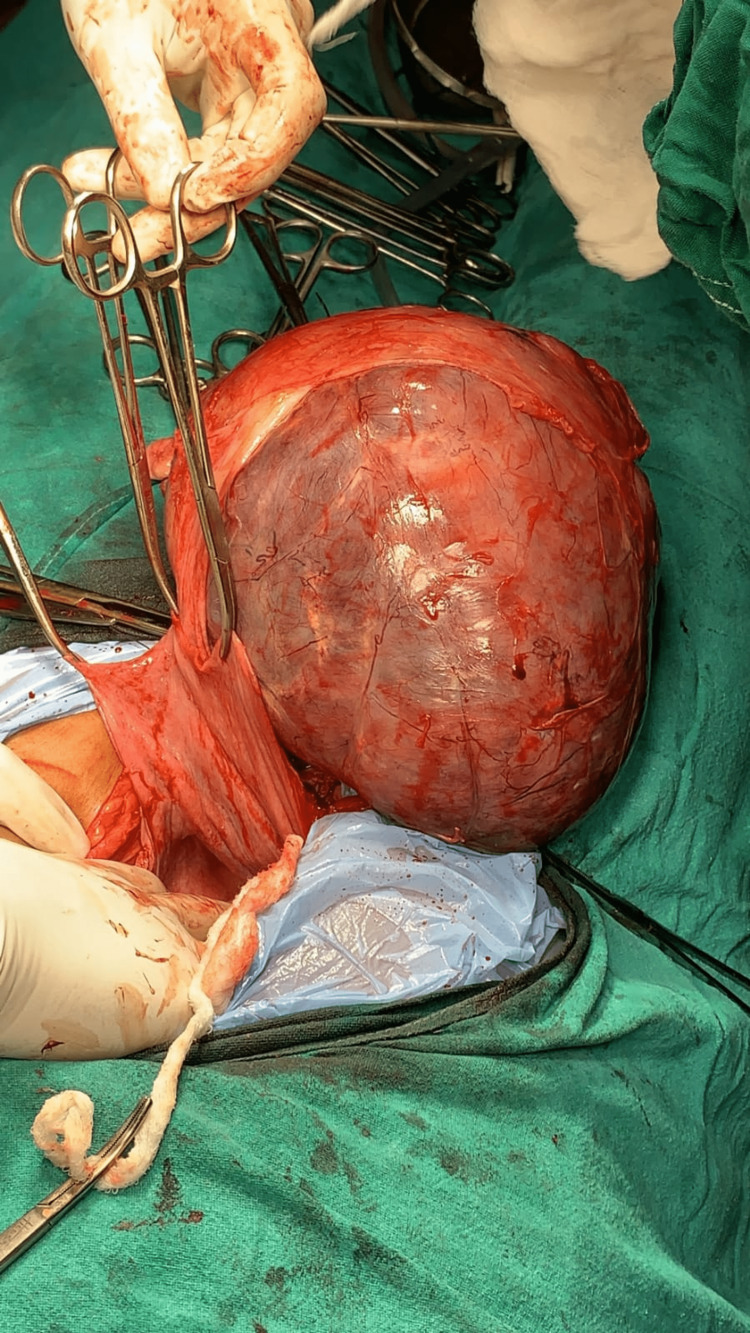
An intraoperative image of the cervical fibroid getting decapsulated.

Prior to the hysterectomy, the ureters on each side were dissected and maintained under observation during the procedure. Without any intraoperative problems, the whole enormous cervical fibroid, measuring 18.5x15x10 cm and weighing 2.4 kg, was first decapsulated from the cervix (Figure 4).

**Figure 5:**
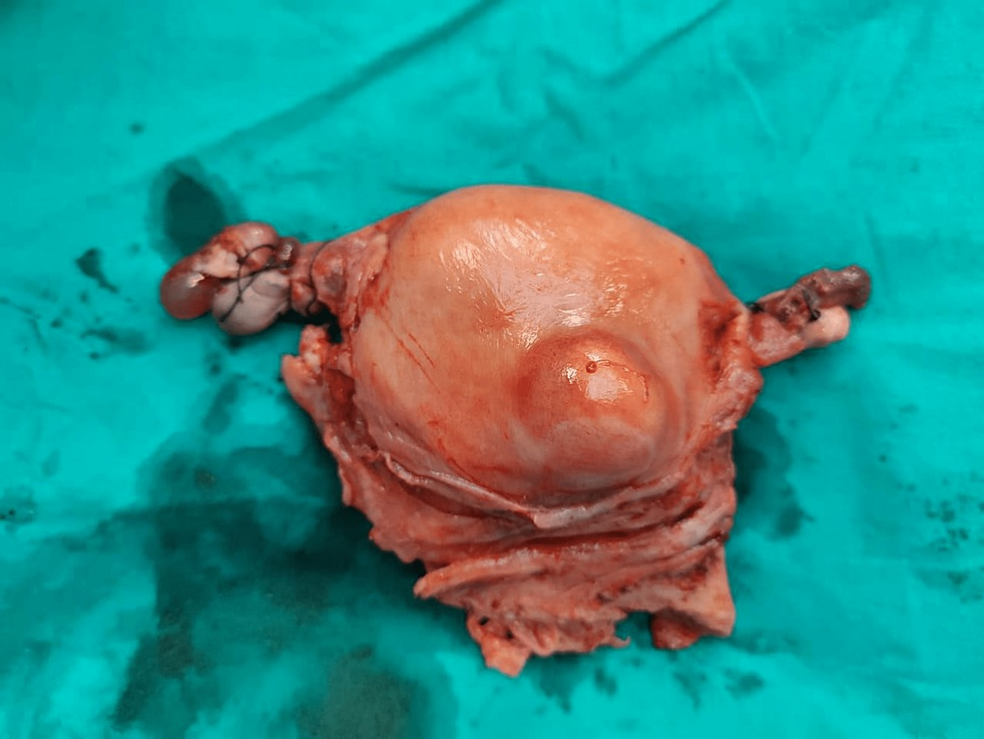
A specimen image of the uterus with a small anterior wall intramural fibroid with bilateral fallopian tubes and ovaries without the cervical fibroid.

**Figure 6:**
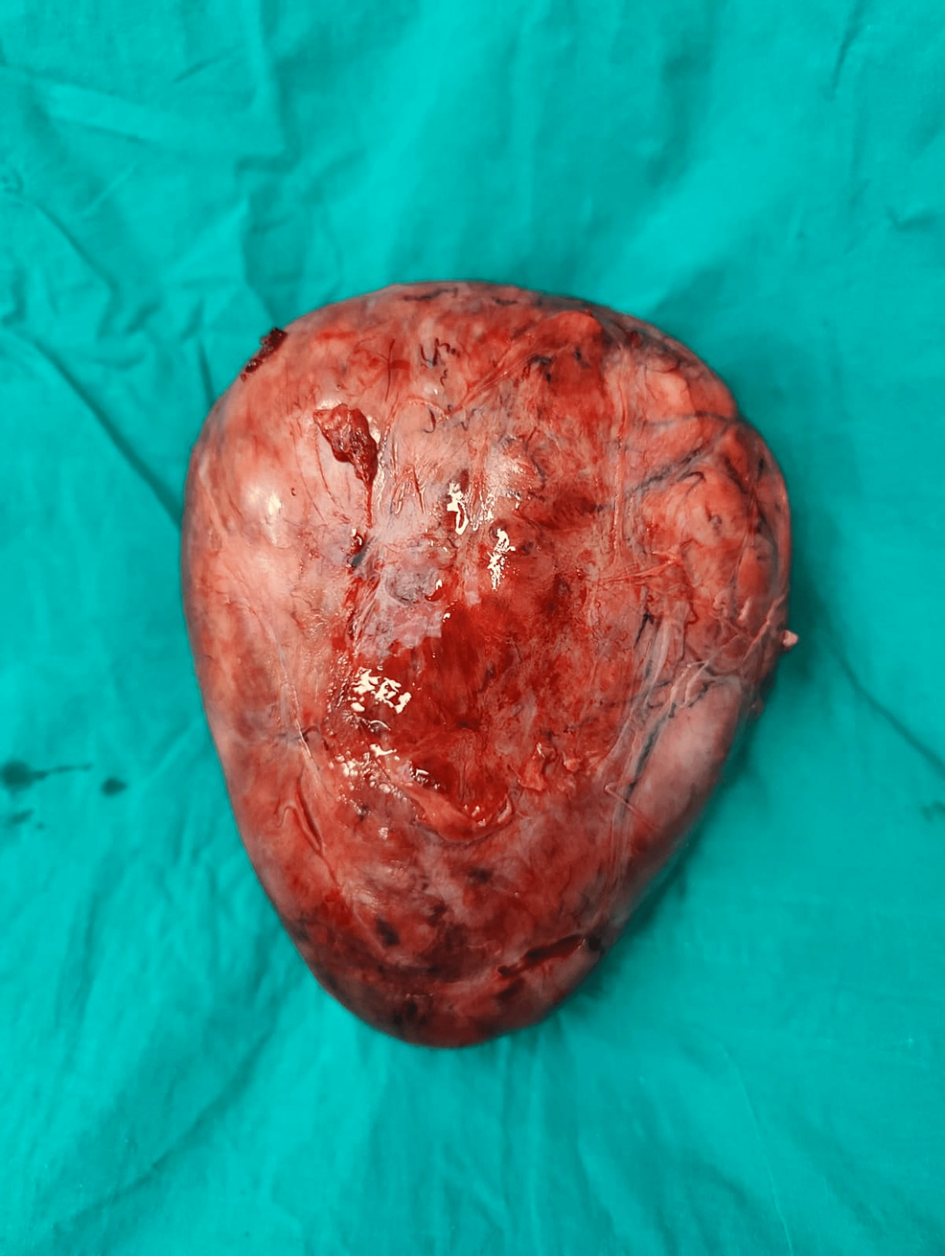
A postoperative image of the decapsulated cervical fibroid.

Next, the uterus, along with the bilateral ovaries and fallopian tubes, were removed in their entirety (Figure 5 and Figure 6). 

The recovery process was uneventful. The diagnosis of leiomyoma was confirmed by histopathology.

## Discussion

Although a fibroid is the most common pelvic tumor in women of reproductive age (20%), a cervical fibroid occurs in only 1%-2% of women [5]. A cervical fibroid may form in the vaginal or supravaginal portions of the cervix, but it often does so in the latter. Depending on where it is located, each fibroid presents differently. The most common complaints at the time of presentation are urinary retention, constipation, and menstrual abnormalities; rarely, it may present as a palpable mass in the abdomen and be misdiagnosed as an ovarian tumor also [6]. Imaging techniques including USG, computed tomography (CT) scans, and MRI may accurately distinguish between uterine and cervical masses. But diagnostic errors can happen in the identification of solid masses. Preoperative uterine embolization helps to reduce the blood flow in the uterus and subsequently reduces blood loss when operating the uterus during surgery, which is an advantage to a temporary uterine artery ligation and reduces the need for intra-operative and post-operative transfusions [7]. Atrophy of small, potential residual fibroids after a uterine artery embolization (UAE) is another result of long-term decreased blood supply. Ngeh et al. [8] reported a significantly lower intraoperative blood loss in 5 patients after embolization (100 mL) versus 14 patients without embolization (400 mL; p = 0.026). In the work of Tixier et al. [9], there was also a significantly lower intraoperative blood loss for patients with versus without UAE. According to some reports, UAE causes less intraoperative blood loss than fibroid enucleation alone, which, for us, was and still is a good enough reason to perform it before a hysterectomy. Prior identification of the ureters with either retroperitoneal dissection or pre-procedure cystoscopic ureteric stenting may be of help in selected cases of very large cervical fibroids with lateral projection [10]. The probability of ureteric injuries in gynecological surgeries of the pelvic cavity increases by 0.2-1.6% [11,12]. In a case of a large cervical fibroid (30×14×10 cm), reported by Basnet et al. [13], bladder injury was noted during surgery. To establish hemostasis, they also had to ligate both internal iliac arteries. Latrogenic ureteric damage not only hurts a patient's physical and emotional health but also raises the possibility of doctor-patient conflicts. Thus, an ability to discriminate and avoid the occurrence of ureteric injury is within our purview. As a result, it is possible to distinguish between different scenarios and prevent ureteric damage from happening.

## Conclusions

The secret to a favorable outcome in cases of massive cervical fibroids is anticipating difficulties and taking preventative action before and during surgery. In our case, the multi-disciplinary preoperative management of the foreseeable complications was fruitful, as intraoperative blood loss was minimal and ureters were protected from iatrogenic injury. The most important lesson to take from this situation is to be on the lookout for potential difficulties. Thus the proverb "prevention is always better than cure" is aptly applicable in this case.

## References

[1] Tiltman AJ (1998). Leiomyomas of the uterine cervix: a study of frequency. Int J Gynecol Pathol.

[2] Jeffcoate N (2001). Tumors of corpus uteri. Jeffcoate’s Principles of Gynaecology, 6th edition.

[3] Stamatopoulos CP, Mikos T, Grimbizis GF, Dimitriadis AS, Efstratiou I, Stamatopoulos P, Tarlatzis BC (2012). Value of magnetic resonance imaging in diagnosis of adenomyosis and myomas of the uterus. J Minim Invasive Gynecol.

[4] Subramaniam R, Vijayananthan A, Omar S, Nawawi O, Abdullah B (2010). Uterine artery embolisation for symptomatic fibroids: the University of Malaya Medical Centre experience. Biomed Imaging Interv J.

[5] Kaushal A, Kaur M, Bhalla V, Kaur D (2018). Unusual giant central cervical leiomyoma: surgical challenge. Int J Reprod Contracept Obstet Gynecol.

[6] Usha Kiran TS, Katke RD, Pimple P (2016). Large cervical fibroid. J Case Rep.

[7] Wu H, Kaczmarski K, Portnoy E, Wang K, Simpson K, Patzkowsky K (2019). 1493 preoperative uterine artery embolization prior to the surgical management of fibroids: an institutional case series. J Minim Invasive Gynecol.

[8] Ngeh N, Belli AM, Morgan R, Manyonda I (2004). Pre-myomectomy uterine artery embolisation minimises operative blood loss. BJOG.

[9] Tixier H, Grevoul J, Loffroy R (2010). Preoperative embolization or ligature of the uterine arteries in preparation for conservative uterine fibroma surgery. Acta Obstet Gynecol Scand.

[10] Patel P, Banker M, Munshi S, Bhalla A (2011). Handling cervical myomas. J Gynecol Endosc Surg.

[11] Tanaka Y, Asada H, Kuji N, Yoshimura Y (2008). Ureteral catheter placement for prevention of ureteral injury during laparoscopic hysterectomy. J Obstet Gynaecol Res.

[12] Han L, Cao R, Jiang JY, Xi Y, Li XC, Yu GH (2014). Preset ureter catheter in laparoscopic radical hysterectomy of cervical cancer. Genet Mol Res.

[13] Basnet N, Banerjee B, Badani U (2005). An unusual presentation of huge cervical fibroid. Kathmandu Univ Med J (KUMJ).

